# Fibulin-1 Is Downregulated Through Promoter Hypermethylation in Colorectal Cancer: A Consort Study

**DOI:** 10.1097/MD.0000000000000663

**Published:** 2015-04-03

**Authors:** Zhiying Xu, Hui Chen, Deliang Liu, Jirong Huo

**Affiliations:** From the Department of Gastroenterology (ZX, DL, JH), 2nd Xiangya Hospital, Central South University, Changsha, Hunan; and Department of Gastroenterology (ZX, HC), People's Hospital of Taizhou, Taizhou, Jiangsu, China.

## Abstract

Fibulin-1 (FBLN1) is involved in the progression of some types of cancer. However, the role of FBLN1 in colorectal cancer (CRC) has not been examined. The purpose of this study was to understand the molecular mechanisms and clinical significance of FBLN1 inactivation in CRC.

The expression of FBLN1 in CRC tissues and adjacent normal tissues was analyzed by immunohistochemical analysis and quantitative real-time polymerase chain reaction (qRT-PCR). Methylation-specific polymerase chain reaction (MSP) and bisulfite sequencing PCR (BSP) were performed to examine the methylation status of the *FBLN1* gene promoter. Furthermore, the methylated level of FBLN1 was analyzed with the clinicopathological characteristics.

Immunohistochemical analysis and qRT-PCR analysis showed that FBLN1 protein and messenger RNA (mRNA) levels in tumor tissues were both significantly decreased compared with that in adjacent nontumor tissues. The methylation rate of FBLN1 promoter was significantly higher in CRC tissues than that in adjacent nontumor tissues (*P* < 0.001). In addition, the correlation between FBLN1 hypermethylation, protein expression, and overall survival (OS) was statistically significant.

Our results indicated that the *FBLN1* gene may be a novel candidate of tumor suppressor gene in CRC, and that promoter hypermethylation of FBLN1 is an important reason for its downregulation and is also a good predictor of OS for CRC.

## INTRODUCTION

Colorectal cancer (CRC) is the third most common cancer in the world and is the fourth cause of cancer death. Despite recent advances in detection and therapy, 25% of these patients will develop metastasis and have a very low 5-year survival rate of around 10%.^1^ The genetic aberrations (such as genetic mutations and chromosomal deletions, etc) as well as epigenetic alterations (such as DNA methylation and histone modifications, etc) have been reported to be involved in the development of CRC.^2^ To date, the most widely studied aberrant epigenetic mechanism is DNA methylation, which occurs in cytosine-phosphate-guanine (CpG)-rich clusters known as CpG islands in regulatory regions of many genes.^3,4^ Inactivations of tumor suppressor genes (TSGs) resulting from DNA hypermethylation play an important role in the formation and procession of CRC.^5^ Moreover, DNA hypermethylation of TSGs has also been proposed as a novel biomarker for detecting tumor and predicting prognosis.

Fibulin 1 (FBLN1) belongs to a growing family of extracellular glycoproteins with distinctive features of a fibulin-type C-terminal domain preceded by tandem epidermal growth factor-like modules.^6^ FBLN1 was reported to be downregulated and function as TSG in many kinds of cancers, such as gastric cancer, prostate cancer, breast cancer, and so on.^7–12^ Studies revealed that downregulation of FBLN1 is due to its promoter hypermethylation in many tumors.^7,8,11,12^ However, the expression levels of FBLN1 in CRC and its possible regulation mechanism remain unknown.

In the present study, methylation-specific polymerase chain reaction (MSP) and bisulfite sequencing PCR (BSP) were performed to examine the methylation status of the *FBLN1* gene promoter. The FBLN1 protein levels in CRC tissues were detected by immunohistochemical analysis to determine whether its promoter hypermethylation correlated with protein downregulation in CRC. Furthermore, regular follow-up of these patients and correlation of molecular findings with disease outcome underscored the prognostic relevance of FBLN1 hypermethylation in CRC patients.

## METHODS

### Clinical Tissue Samples

Specimens of cancer tissues and adjacent normal tissues from 68 CRC patients were obtained from Xiangya Second Hospital, Central South University, Hunan, China, during the period between January 2008 and October 2009. Tumor samples were diagnosed according to the World Health Organization (WHO) system, by two pathologists who were unaware of patient data. Patients who had undergone radiotherapy or chemotherapy prior to surgery were excluded from the study. Clinical data of the patients, including gender, age, tumor differentiation, lymph node metastasis, clinical grade, were obtained from the hospital records, which are shown in Table 1. The patient group comprised 46 men and 22 women. The patient ages ranged from 35 to 72 years, with an average of 59.8 years. The tumor was in stage III/IV in 48.5% (33/68) of patients and the differentiation grade was poor or no in 42.6% (29/68). 44.1% (30/68) of patients had lymph node metastasis. These patients were followed up for a period of nearly to 5 years. Overall survival (OS), defined as the time from the date of diagnosis to the date of death or last contact if the patient was still alive, ranged from 6 to 54 months (median, 32 months). The study was approved by the human ethics committee of the Central South University. Informed consent was obtained from all subjects studied.

**TABLE 1:**
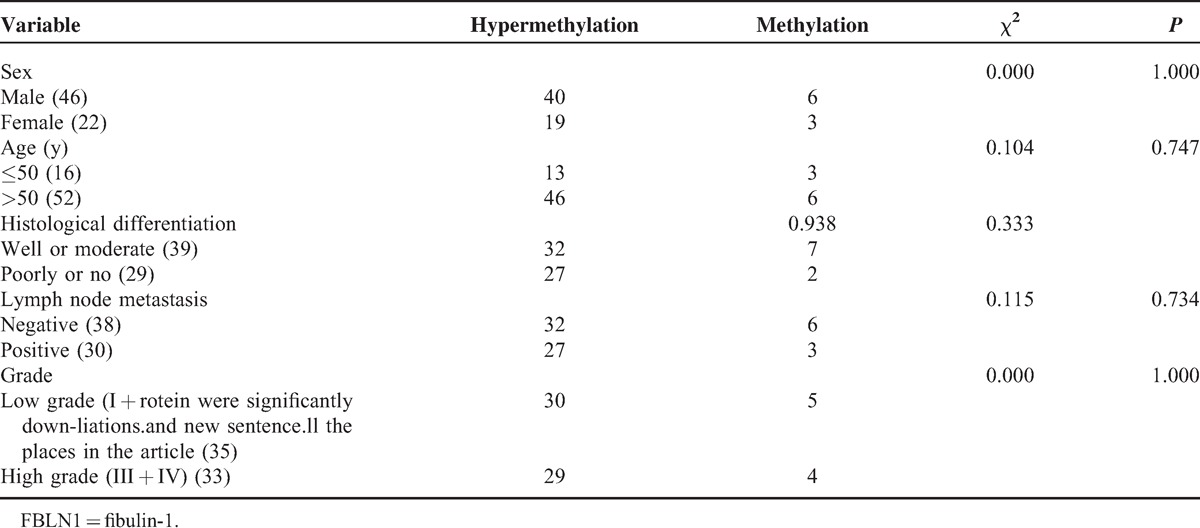
Correlation of FBLN1 Hypermethylation With Clinicopathological Characteristics

### DNA Extraction and Bisulfite DNA

Genomic DNA was isolated from CRC tissues and adjacent normal tissues by use of the Universal Genomic DNA Extraction Kit Ver.3.0 (Takara, Dalian, Liaoning, China) according to the manufacturer's instructions. The quality and integrity of DNA were estimated by electrophoresis on 1% agarose gel.

Bisulfite modification of DNA was produced using an Epitect Bisulfite Kit (Qiagen, Hilden, Germany) as recommended by the manufacturer. Briefly, genomic DNA (1 μg) was denatured using NaOH, and then subjected to sodium bisulfite treatment. Subsequently, the mixture was desulfonated, and the DNA was purified on silica-membrane columns to a final volume of 20 μL. The bisulfite-modified DNA was stored at −20 °C for the further use.

### Methylation-Specific PCR

Bisulfite-modified DNA was amplified with primers specific for methylated or unmethylated sequences. The methylated DNA was amplified using M primers: 5′-CGGGGATAATTTTT GATTTC-3′ (sense) and 5′-ACCCCGACTACGAAAAAC-3′ (antisense), and unmethylated DNA was amplified using U primers: 5′- GTGGGGATAATTTTTGATTTT-3′ (sense), and 5′-AACCCCAACTACAAAAAAC-3′ (antisense). MSP for the FBLN1 promoter was conducted in a total PCR volume of 20 μL, and the PCR products were analyzed by 1% agarose gel electrophoresis.

### Bisulfite Sequencing PCR

BSP was conducted as described previously.^13^ The bisulfite-treated FBLN1 promoter containing 36 CpG sites was amplified with the primers 5′- TTTTTGTTTTTGAGGGTAGA GT-3′ (sense) and 5′- ATCTCCTAATCCTCCCTCC-3′ (antisense). The amplified PCR products were purified and subcloned into pGEM T-Easy vector (Promega, Madison, WI). A total of 5 to 10 clones of tumor and adjacent nontumor tissue samples were sequenced. The percentage of methylated CpG dinucleotides was calculated to evaluate the methylation level of FBLN1.

### Immunohistochemistry

Immunohistochemical analysis was carried out using formalin-fixed paraffin-embedded CRC tissue and adjacent normal tissue. In brief, after antigen retrieval, tissue sections were incubated with primary FBLN1 antibody (1:100 dilution, Santa Cruz, CA) at 4°C overnight. After washing with PBS, tissue sections were incubated with biotin-linked universal antibodies and then with horse-radish peroxidase–streptavidin conjugate. And the sections were incubated with 3, 3′-diaminobenzidine hydrochloride (DAB). The tissue sections were observed by using microscope (OLYMPUS BX-51, Japan). The primary antibody was replaced by PBS as a negative control. The slides were evaluated independently in a blinded manner by two observers, one of whom is a professor of Pathology of Xiangya Second Hospital, Central South University. The FBLN1 staining of cells was scored for intensity (0, 1+, 2+, 3+) and percentage of staining (1, 0%–25%; 2, 26%–50%; 3, 51%–75%; 4, 76%–100%). The sum of intensity and percentage counts was used as the final score. For the statistical analysis, FBLN1 expression was considered in two categories: low (<8) or high (≥8).

### RNA Isolation and Quantitative Reverse-Transcriptase Polymerase Chain Reaction

Quantitative reverse-transcriptase polymerase chain reaction (qRT-PCR) was performed to determine the expression of FBLN1 mRNA in tumor and normal tissues. Briefly, total RNA was extracted from frozen tissues with Trizol Reagent (Applied Invitrogen, Carlsbad, CA) according to the manufacturer's protocol. The levels of FBLN1 messenger RNA (mRNA) were quantified with the Applied Biosystems 7900HT Fast Real-Time PCR System using SYBR Premix Ex Taq (Takara, Dalian, China). GAPDH was used as an internal control. The sequences of the FBLN1 primers were as follows: forward 5′-TGCGAATGCAAGACGG-3′ and reverse 5′-CG TAGACGTTGGCACA-3′.

### Statistical Analysis

The difference of FBLN1 promoter methylation status between CRC tissues and adjacent normal tissue was examined by Student test. The relationships between FBLN1 methylation status, protein expression, and clinicopathological parameters were examined by χ^2^ test. Survival curves were estimated by the Kaplan–Meier method, and the differences in OS between subgroups were compared by the log-rank test. Results were regarded as significant when *P* ≤ =0.05.

## RESULTS

### FBLN1 Hypermethylation in CRC Tissues

**FIGURE 1:**
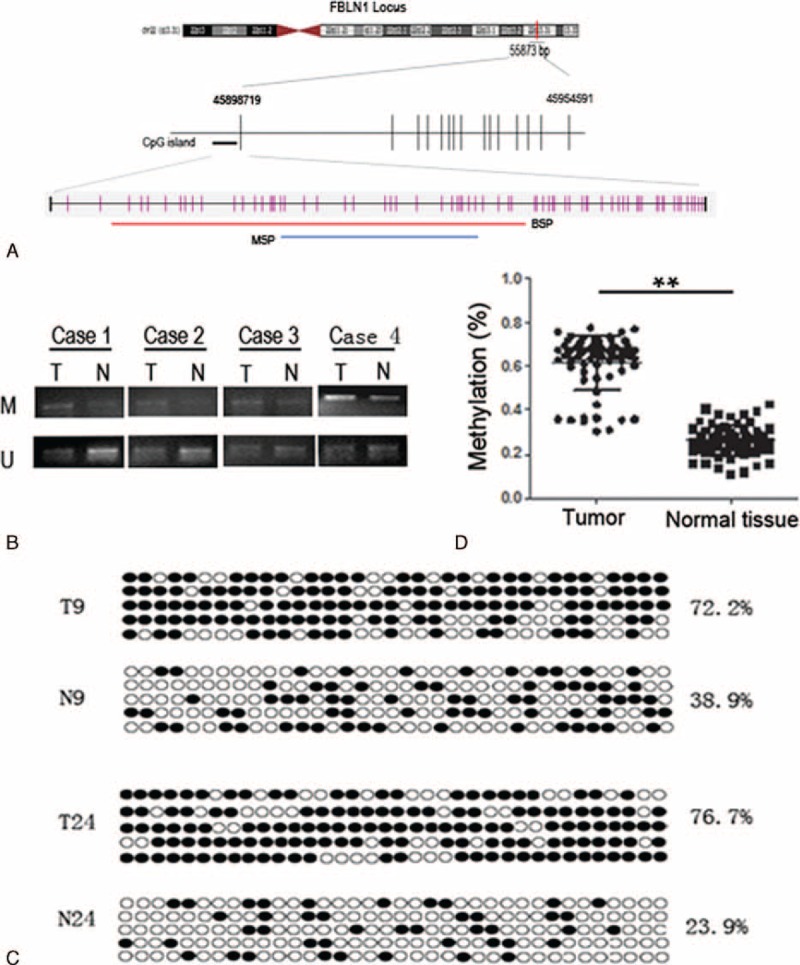
Determination of FBLN1 methylation status. (A) Schematic diagram of CpG dinucleotides within the FBLN1 promoter. FBLN1 is located on chromosome 22, band q13. The promoter region contains a CpG island. CpG sites are shown as pink bars. The blue line shows the region tested in MSP, and the red line indicates the region tested in BSP. (B) Representative results of the MSP analysis for FBLN1 in selected four pairs of samples, respectively. (U = unmethylated; M = methylated; T = tumor tissue; N = adjacent normal tissue.) (C) Bisulfite sequencing analysis of the upstream regulatory region of FBLN1 in representative tissues (N = adjacent nontumor tissue; T = CRC sample). For each sample, at least five separate clones were sequenced and the results are shown here. Black and white circles represent methylated and unmethylated CpG, respectively. For each row of circles sequence results for an individual clone of the bisulfite-PCR product are given. The methylation level is given as a percentage on the right of each bisulfite result. (D) The methylation status of FBLN1 promoter was compared between CRC tissues and adjacent nontumor tissues. (BSP = bisulfite sequencing polymerase chain reaction; CpG = cytosine-phosphate-guanine, FBLN1 = fibulin-1; MSP = methylation-specific polymerase chain reaction; PCR =  polymerase chain reaction). ^∗∗^*P* < 0.001.

To investigate whether epigenetic alteration could be extrapolated to CRC, we firstly performed MSP analysis to determine the methylation status in the promoter regions of FBLN1 in CRC tissues and adjacent nontumor tissues. The results showed that the FBLN1 promoter was hypermethylated in tumor tissues (Figure 1A and 1B). Furthermore, we designed and validated bisulfate sequencing PCR for FBLN1 methylation within its promoter region which included 36 CpGs (Figure 1A). The results showed that the CpG sites were highly methylated in tumor tissues for FBLN1, and the methylation level varied from 30% to 77.2%, with a mean ratio of 61.54% in the tumor tissue (Figure 1C and 1D). In contrast, the methylation level observed in the adjacent nontumor samples ranged from 10.6% to 42.8%, with a mean of and 25.98% (Figure 1C and 1D). Methylation levels >36.8% were considered hypermethylation. FBLN1 was hypermethylated in 59 (86.8%) of 68 tumors.

### Correlation of FBLN1 Hypermethylation With Clinicopathological Characteristics

For some tumors, hypermethylation of satellite DNA sequences has been significantly correlated with malignant potential, aggressive histological features, and worse prognosis. Accordingly, we evaluated the methylation status of the FBLN1 promoter in 68 CRC samples using BSP. Most (59/68, 86.8%) of these samples were hypermethylated compared with the adjacent nontumor tissues. Unfortunately, there was no statistically significant correlation between sex, age, histological differentiation, lymph node metastasis, grade, and FBLN1 hypermethylation (Table 1).

### Correlation of FBLN1 Hypermethylation With Its Expression Levels

**FIGURE 2:**
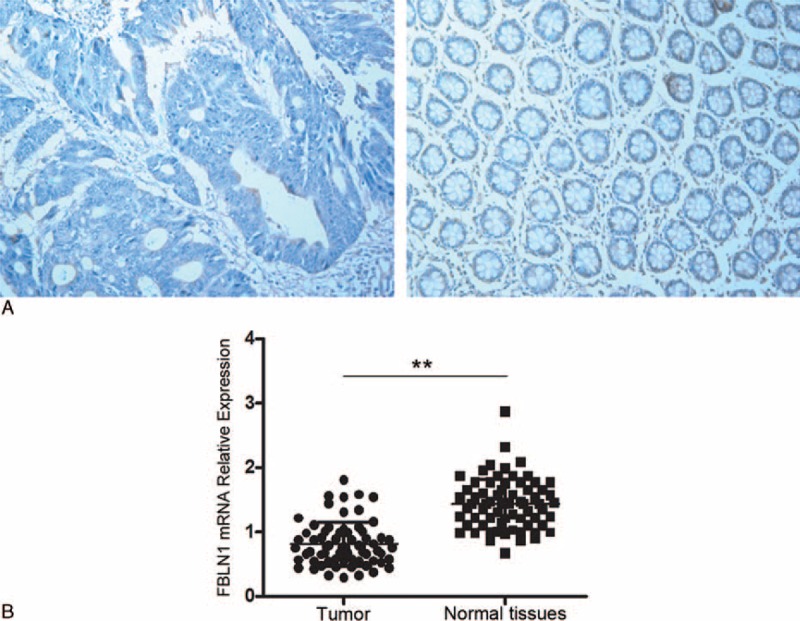
Determination of FBLN1 expression levels. (A) Immunohistochemical analysis of FBLN1. Paraffin-embedded tissue sections from CRC and adjacent normal tissues were used for immunohistochemical analysis of FBLN1 protein with monoclonal antibody of FBLN1. The photomicrograph shows a CRC sample (left) showing low-level expression of FBLN1, and an adjacent nontumor tissue (right) showing high-level expression. (B) The relative mRNA levels of FBLN1 were compared between CRC tissues and adjacent nontumor tissues. (CRC = colorectal cancer; FBLN1 = fibulin-1; mRNA = messenger RNA). ^∗∗^*P* < 0.001.

We measured the expression of FBLN1 by immunohistochemical analysis in 68 pairs of tumor and adjacent nontumor tissues. As shown in Figure 2A, the expression levels of FBLN1 protein were significantly downregulated in tumor tissues compared with nontumor tissues. Decreased expression of FBLN1 protein in 49 (72%) of 68 CRC tissues was shown (Table 2). We also determined the FBLN1 mRNA levels by qRT-PCR. The result showed that the FBLN1 mRNA levels were also decreased in CRC, compared with that of normal tissues (*P* < 0.001, Figure 2B). Comparison of methylation data with immunohistochemistry findings revealed low-level or loss of FBLN1 protein expression in 48 (81.4%) of the 59 tumors harboring FBLN1 hypermethylation. In contrast, increased expression of FBLN1 protein was observed in 8/9 (88.9%) of low-level methylated tumors and reduced expression was only observed in 1/9 (11.1%) (Table 2). There was a significant association between FBLN1 promoter hypermethylation and its protein expression (*P* < 0.001, Table 2).

**TABLE 2:**
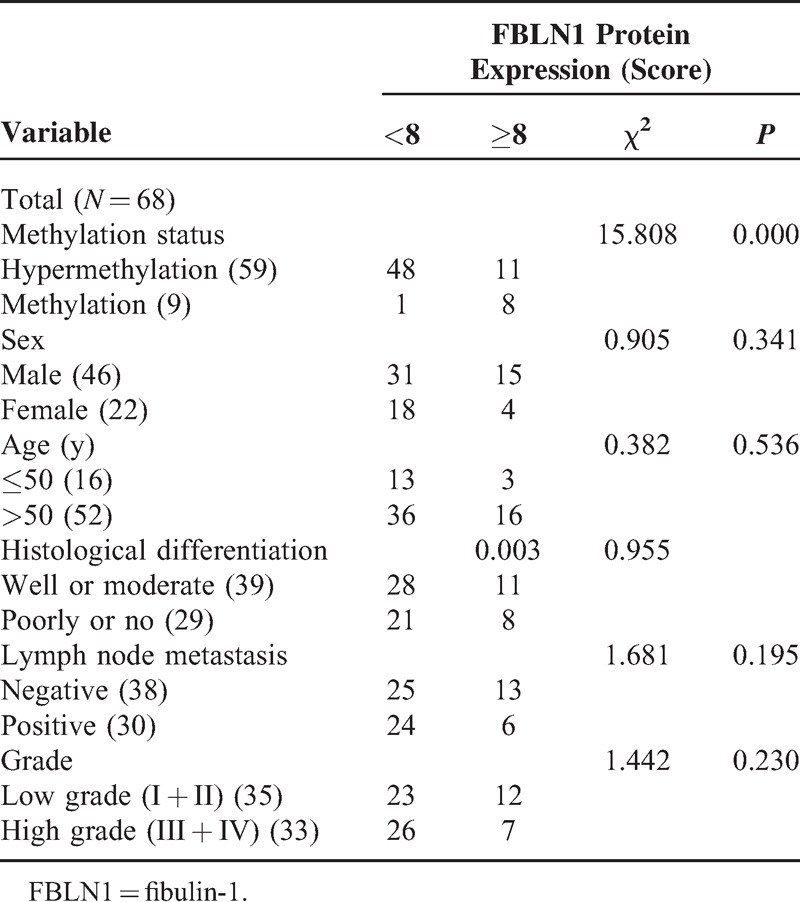
Association of Protein Expression With FBLN1 Hypermethylation and Clinicopathological Characteristics

### Comparisons of Protein Expression With Clinicopathologic Characteristics of Patients

We also compared the expression of FBLN1 protein in CRC samples with different clinicopathologic characteristics including sex, age, histological differentiation, lymph node metastasis, clinical grade. However, no significant difference was found between subgroups of patients (Table 2).

### Clinical Outcome of the Patients

**FIGURE 3:**
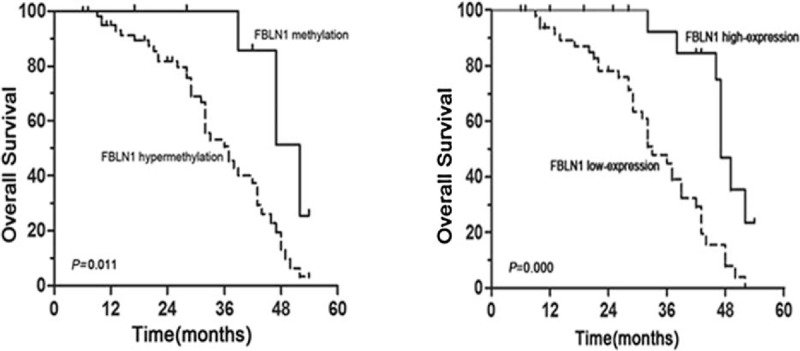
Kaplan–Meier analysis of overall survival in relation to FBLN1 methylation status and FBLN1 protein expression. (A) The correlation between FBLN1 methylation in the tumor tissue and the OS of the CRC patients. The patients with hypermethylation of FBLN1 had a shorter OS than those with normal levels. (B) The correlation between FBLN1 protein expression in the tumor and the OS of the CRC patients. The patients with a low level of FBLN1 expression had a poor outcome. (CRC = colorectal cancer; FBLN1 = fibulin-1; OS = overall survival).

To investigate the prognostic value of FBLN1 hypermethylation status and FBLN1 protein expression for CRC, we assessed the association between FBLN1 methylation status or protein expression and survival duration by using the Kaplan–Meier analysis. CRC patients with hypermethylated FBLN1 promoter in tumors revealed shorter OS as compared to patients without hypermethylated promoter (*P* = 0.011, Figure 3A). Similarly, the correlation between FBLN1 protein expression and OS was statistically significant; markedly reduced OS was observed in CRC patients having FBLN1 low expression (*P* = 0.000, Figure 3B).

## DISCUSSION

DNA methylation of promoter CpG islands has been recognized as an important mechanism for regulation of gene expression and transcriptional modification in mammals. Alteration in DNA methylation patterns may have critical effects on tumor initiation and progression.^14^ Epigenetic alterations such as promoter hypermethylation can lead to the transcriptional silencing of TSGs, which are important for preventing cancer development.^5^ Given that most, if not all, TSGs can be inactivated through promoter hypermethylation in human cancers, promoter hypermethylation has been recognized as one of the most important markers for identifying novel TSGs.

FBLN1 is located on chromosome 22, band q13, and is a member of a growing family of extracellular glycoproteins.^15^ It had been reported that FBLN1 functions as a TSG and is specifically downregulated in many tumor tissues. FBLN1 is epigenetically silenced in some cancers through promoter hypermethylation.^7,8,11,12^ However, whether FBLN1 functions as a TSG in CRC remains unknown.

In the present study, the result of immunohistochemical and qRT-PCR analyses showed that FBLN1 protein and mRNA expression levels were significantly downregulated in CRC tissues. In our methylation analysis, CRC tissues were significantly hypermethylated with a mean methylation level of 61.54%, compared with adjacent nontumor sample (a mean of 25.98%). In addition, a statistically significant correlation between low-level expression of FBLN1 protein and FBLN1 hypermethylation in CRC was observed. This suggests that promoter hypermethylation is one of the major mechanisms for inactivating FBLN1 in CRC. Furthermore, we focused the correlation between FBLN1 methylation status and the clinicopathological features. Our data indicated that there was no statistically remarkable correlation between sex, age, histological differentiation, lymph node metastasis, grade, and FBLN1 hypermethylation. Kaplan–Meier analysis showed that FBLN1 methylation status was inversely correlated with OS of the CRC patients. These findings suggested that FBLN1 hypermethylation would be an early event in CRC, and FBLN1 inactivation could play an important role in the initiation and formation of CRC. Thus, promoter hypermethylation of the FBLN1 gene would provide a potential biomarker of early prognosis and clinical outcome in patients with CRC.

In summary, our study provides evidence that FBLN1 functions as a novel candidate TSG in CRC, and its downregulation may be due to promoter hypermethylation. Promoter hypermethylation of FBLN1 can be a novel biomarker of early prognosis and clinical outcome for CRC patients.
